# Appendicular and Caecal Fecalith causing Perforation: A Case Report

**DOI:** 10.31729/jnma.4711

**Published:** 2020-04-30

**Authors:** Rojan Adhikari, Prashant Simkhada, Deependra Mandal, Ashok Kunwar, Saroj Prasad Dhital

**Affiliations:** 1 Department of Surgery, Kathmandu Model Hospital, Adwait Marg, Kathmandu, Nepal; 2 Kanti Childrens Hospital, Maharajgunj, Kathmandu, Nepal; 3 Kathmandu Medical College and Teaching Hospital, Sinamangal, Kathmandu, Nepal

**Keywords:** *appendicitis*, *case report*, *cecum*, *fecalith*

## Abstract

Fecalith is a concretion of dry compact feces or hard stony mass of faeces in the intestinal tract. Though appendicular fecoliths are commonly encountered, caecal fecoliths are rare entities. Fecoliths are amenable to conservative management with laxatives and enemas but surgical management prevents recurrence. We present a case of 27 years old male who was diagnosed with acute appendicitis with peritonitis. He was intraoperatively diagnosed as gangrenous and perforated retrocaecal appendix with multiple small fecaliths and a large fecalith on cecum with perforation. Appendectomy and primary repair of caecal perforation done. Histological examination of perforated margin confirmed as an inflammatory lesion.

## INTRODUCTION

Fecalith of the caecum is very rare and may present as a mass in the right iliac fossa which may be mistaken for a carcinoma.^1^ Fecalith, coprolith or stercolith can obstruct the appendix, leading to appendicitis. The pathophysiology of fecalith formation is usually related to slowed peristalsis of the lower GI tract, dehydration and low fiber diet intake. Fecal impaction if left untreated can lead to the hardening of stools and the formation of fecalith. Caecal fecalith causing acute appendicitis has not been reported in the literature.^2^ Here, we present a rare case of giant fecalith causing caecal perforation and with multiple small fecaliths causing appendicular perforation.

## CASE REPORT

A 27 years old male presented in the Emergency Department with a chief complaint of pain abdomen for 4 days and fever for 1 day. Pain abdomen was initially on the paraumbilical region later migrated to right iliac fossa and was localized. Pain was acute in onset, dull in nature, no aggravating and relieving factor and severity of pain is moderate to severe. For last one day pain was generalized. He had a history of nausea and vomiting three episodes which contained food materials. He also complained of fever, the maximum temperature recorded was 101 degrees Fahrenheit not associated with chills and rigor. He had not to passed stool for 4 days but had normal bladder habits. There was no significant medical and surgical past history. On examination, vitals were within the normal range. Per abdomen, examination showed generalized guarding and bowel sound was absent.

**Figure 1.**
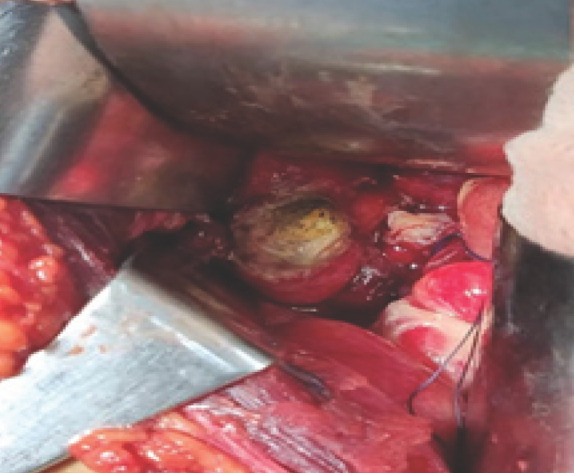
Fecalith in the appendix.

Investigation showed leukocytosis and neutrophilia. USG abdomen and pelvis showed features suggestive of acute appendicitis. Based on the above finding diagnosis of peritonitis secondary to perforated appendicitis was made and planned for exploratory laparotomy with appendectomy. The intraoperative finding was around 250 ml of purulent fluid mixed with fecal content, acutely inflamed gangrenous perforated retrocaecal appendix with multiple small fecalith and perforation of cecum at anterior aspect 2cm distal to the base of the appendix with 2×2 cm fecalith (Figure 1).

**Figure 2.**
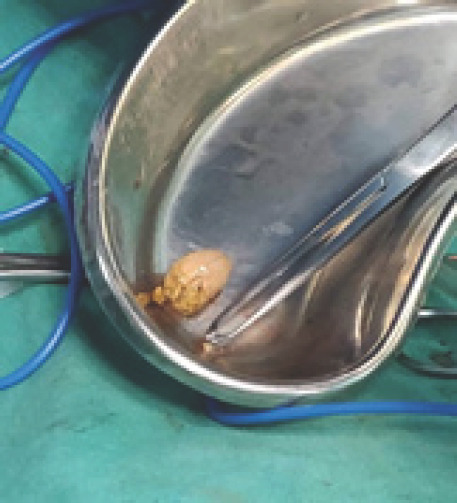
Extraction of fecalith.

Fecolith was extracted (Figure 2) and caecal perforation was repaired with freshening the margin. The postoperative period was uneventful, The patient was managed with analgesics, antacids and antibiotics, and discharged on the fifth postoperative day.

The culture of peritoneal fluid showed Pseudomonas Aeruginosa. Histopathology of the perforated margin of cecum and appendix showed inflammatory features.

## DISCUSSION

The first detailed description of an appendicolith was by Wegeler in 1813.^2^ If the accumulation of faecal matter is slow and desiccation is fast then the mass becomes hard and dry and is called a fecalith.^3^ Appen- dicoliths (also known as faecaliths, coproliths, enteroliths, and stercoliths) are descrete, hard but crushable, mostly ovoid, brown concretions within the appendix; They are often concentrally laminated and frequently calcified.^4,5^ Our case illustrates that fecalith can have an extremely rare presentation in the caecum. The fecalith caused the intestinal obstruction which explains not passing stool in our patient. An increase in intra-abdominal pressure caused by the fecalith decreases capillary perfusion and can lead to ischemic necrosis.^6^ This explains the inflamed gangrenous perforated retrocaecal appendix and perforated caecum.

This case is an adult man with fever and pain in right iliac fossa who was diagnosed as acute appendicitis with peritonitis. Caecal fecalith might have obstructed the appendix orifice and resulted in acute appendicitis. Peritonitis resulted due to perforated appendicitis. Perforation of caecum is an uncommon differential diagnosis for an acute appendicitis.^7^ Blotner reported a case of perforation of cecum caused by a fecalith trapped in a cecal haustration.^8^ Walker reports two cases of caecal fecalith presenting as a mass in the right iliac fossa and found audible borborygmi in his case but in our case bowel sound was absent.^3^ An unusual case of caecal perforation in a child due to caecal fecalith was reported by Simpkin and Lakhoow in which they found perforated caecum but normal appendix.^9^ However in our patient we found perforated caecum and perforated appendix.

In our case we refreshed the margin of wound and sutured it. The treatment of our patient similar treatment done by Simpkin and Lakhoo.^9^

Although the fecalith of the caecum is rare, it has to be considered in the differential diagnosis of the right iliac fossa pain. The complication of the fecalith of the caecum can lead to iliocolic intussusception.^1^ This complication challenges the surgeon because of the wide range of issues that need to be considered for establishing the etiology and therapeutic strategy.^10^ So Fecalith of the caecum has to be surgically removed as it cannot be removed by conservative measures.

## Consent:

**JNMA Case Report Consent Form** was signed by the patient and the original article is attached with the patient's chart.

## Conflict of Interest

**None.**
